# Parent Knowledge and Perceptions of Concussion Related to Youth Football

**DOI:** 10.7759/cureus.2268

**Published:** 2018-03-04

**Authors:** Brian Rieger, Lawrence Lewandowski, Heather Potts, Kyle Potter, Lawrence S. Chin

**Affiliations:** 1 Physical Medicine and Rehabilitation, SUNY Upstate Medical University; 2 Department of Psychology, Syracuse University; 3 Department of Neurosurgery, SUNY Upstate Medical University

**Keywords:** concussion, pediatric, sport, parent, education, youth football

## Abstract

Introduction

There is increased concern about concussion in youth athletes, yet there is little research on parent knowledge of concussion.

Purpose

The purpose of the current study was to investigate attitudes to and knowledge of concussion among parents of youth football players.

Methods

We surveyed 180 parents/guardians of youth football players, ages 5-12, regarding their knowledge and beliefs concerning concussion.

Results

We found that the vast majority of respondents (86%) had confidence in their ability to recognize concussions. Yet, a significant number also held misconceptions about concussions, such as ‘too much sleep’ (48%) or ‘eating certain foods’ (26%) make concussion symptoms worse. Most (82%) had not heard of the Zurich guidelines, and less than half (44%) were aware that sustained mental activity could worsen symptoms. Parents were concerned about their child sustaining a concussion, but a substantial minority also reported ‘serious concern’ about their children losing playing time or their position.

Discussion

Results are somewhat positive in terms of parents’ general knowledge of concussions; yet, response variability and misconceptions point to a continued need for concussion education for parents. Medical professionals can play an important role in informing families about concussion symptoms, management, and recovery.

## Introduction

According to an Entertainment and Sports Programming Networks (ESPN) poll, parents now cite concussion as the injury they most worry about in their children playing youth sports [1]. These concerns arise in part from increased media attention to concussions, as well as high-profile case studies of many professional athletes suffering the aftereffects of head trauma. Despite increased attention to high-profile athletes and new concussion management protocols for college and professional sports, there is still only limited research into the actual numbers of concussions suffered in younger athletes, particularly those playing nonscholastic youth sports. Emergency department data from 2001-2005 reported that for youth, ages 8-13, 58% of concussions are due to organized team sports, with football accounting for 37% of those [2]. In 2011 alone, 55,000 high school football players and 29,000 youth soccer players in the US sustained a concussion [3]. The number of athletes age 13 and younger who suffer sports-related concussions but do not present to an emergency department is unknown, and many concussions may not be recognized or reported [4]. In a recent study of concussion in youth football, Kontos and colleagues [5] reported that the rate of concussion in football practice for 8-12-year olds' is similar to high school and college players, but the game rate is at least twice as high. Forty-five percent of concussions involved head-to-head contact, and 11-12-year olds' were 2.9 times more likely to suffer a concussion than 8-10-year olds were.

Some experts have argued that the accumulation of blows to the head, rather than a significant concussive event, may be more damaging to the brain and produce negative functional outcomes [6]. Various investigators have examined the number and magnitude of head impacts absorbed by children playing youth football [7-9]. They concluded that while head impact forces are lower for younger athletes, as opposed to high school and college athletes, they can, at times, suffer the same impact forces as older students, including high magnitude impacts. It appears that public awareness of concussions and potential long-term effects of accumulative head impacts are influencing some parents’ decisions about letting their children play football. A Wall Street Journal poll [10] found that 40% of parents would dissuade their children from playing football. Although no causal relationship has been established, statistics from Pop Warner youth football leagues indicate recent declines in participation [10]. We can only presume that this may be due, in part, to the increasing awareness about concussion.

As it pertains to professional sports, the consequences of concussion have garnered an increased amount of attention. A study by Stern and colleagues [11] found that National Football League (NFL) players who started playing football before age 12 had significantly worse cognitive and behavioral symptoms than their counterparts who started playing after 12 years of age. Other research has shown that sports concussion likely increases the risk for dementia [12] and depression [13] later in life. There is also accumulating evidence to suggest that repeated subconcussive blows can cause structural injury to the brain [14], and repeated head trauma in athletes has been implicated in a condition known as chronic traumatic encephalopathy [11]. Findings such as these have further fueled concerns about concussion in youth sports and have spurred efforts to mitigate risk. Pop Warner football recently implemented new guidelines to reduce the amount of contact allowed in exercise drills [15] with a corresponding reduction in concussions [16]. Concerns about sports concussion have also led to an increased demand for appropriate concussion management. As of 2014, all 50 states and the District of Columbia have passed Return-to-Play laws that cover concussions in youth and high school athletes [17].

There are still many unknowns regarding the long-term risks of concussion or blows to the head suffered in the context of football or other sports, but there is a consensus among experts as to what constitutes appropriate concussion management. This involves (a) recognizing a concussion when it occurs, (b) immediately removing the athlete from play after a concussion, and (c) ensuring the athlete is fully recovered before returning to play [13]. State concussion laws have attempted to codify these recommendations by mandating concussion education, immediate removal of an athlete from play or practice in the event of a suspected concussion, and a requirement that the athlete not return to play until cleared by a professional with expertise in concussions. Although all 50 states require parents and youth athletes to complete yearly concussion education training, there is considerable variability across states as to the education and certification of coaches, athletic staff, and sports volunteers [17].

In non-scholastic youth sports, the burden of recognizing a concussion falls primarily on coaches and parents. The Centers for Disease Control’s (CDC) ‘Heads-Up’ campaign is aimed at better educating athletes, parents, coaches, and school personnel about concussion [18], but little is known about current knowledge and attitudes among these groups. Mannings and colleagues [19] developed a survey based on the CDC materials and administered it to 310 parents of youth football players (ages 5-15). They found that most parents did not know that a concussion is a mild traumatic brain injury. None could correctly classify an entire list of symptoms as concussion-related or not. The author concluded that parents had some knowledge of concussion but that various misconceptions remained.

Research on concussion knowledge has focused on coaches as well as parents. A recent meta-analysis [20] revealed that knowledge of concussions among coaches and parents was marginal at best. Across studies, coaches cited the misconception that there needed to be a loss of consciousness to indicate a concussion, which is not true. Additionally, a quarter of coaches reported that they would not remove a player if they suspected a concussion. Almost one in five parents reported that they would not seek medical attention for a concussion and 4% would allow a symptomatic child to return to play. Chrisman and colleagues [21] found that the Washington State concussion law led to improvements in coaches’ but not parents’ knowledge, most likely due to the fact that the law imposes less strict requirements and regulation for parents of youth athletes. More recently, Lin and colleagues [22] found that concussion knowledge in parents of youth athletes increased with income and education level. 

Overall, it appears that attitudes and knowledge about concussion vary quite a bit among parents and coaches. Further, there seem to be certain misconceptions that parents and coaches have about concussion and its management. In order to increase parental knowledge about concussion and appropriate management practices, information programs will need to be developed and disseminated. Determining what to teach parents begins with discerning what they know and believe about concussions. The purpose of the current study was to investigate attitudes to and knowledge of concussion among parents of youth football players, with the hope of being better informed as to how to educate parents about the risks and myths surrounding sports concussion. We focused on parents of young athletes because they are just beginning to experience the risks of head injuries to their children and can play an important role in ensuring the appropriate management of concussion, should one occur.

## Materials and methods

Participants

Parents/guardians were recruited for a survey study of youth football organizations in central New York. The surveys were collected in late summer and early fall prior to the football season’s start. Researchers went to parent meetings and to organized scrimmages and solicited respondents to fill out surveys. Participation in the study was voluntary and all participants read an introductory statement explaining the purpose of the study. The number of athletes in the program was approximately 300, although not all parents attended the organizational meetings. Minimally, our survey participation rate was 61.7%. Five surveys were excluded from the analysis because the ages of the players were above the age cutoff. Across the 180 surveys included in the final analysis, the players ranged in age from seven to 12 (M = 9.54, SD = 1.38) with a range of 1-7 years (M = 3.08; SD = 1.728) of experience playing youth football. One hundred eighty parents/guardians of youth football players completed the surveys, including 125 mothers, 51 fathers, two guardians, and two that did not report a specific relationship. The majority of parents/guardians in the sample identified as Caucasian/White (n = 151; 83.9%), with the remainder self-identified as African American/Black (n = 4; 2.2%), Asian (n = 1; 0.6%), multiracial (n = 1; 0.6%), Hispanic (n = 3; 1.7%), Native American (n = 3; 1.7%), and Arabic (n = 1; 0.6%). The average age of the adults was 38.7 years, and their average years of education was 14.4.

Eleven players received a diagnosis of concussion from a medical professional sometime prior to the study, four of which occurred during football practice or play. Two of these players had experienced multiple concussions. Of the 11 players who had concussions, four reported school absences because of symptoms and two others reported academic difficulties for a period of weeks. In addition, 49 respondents said that their child had been “dinged,” such that they were noticeably slow to get up or wobbly after a collision. Of these 49, 35 respondents said this happened multiple times and 10 indicated they observed it at least five times.

Materials

In addition to demographic information, the survey contained questions regarding parent/guardian knowledge of concussion (e.g., ability to recognize a concussion, when should a child return to play, are you familiar with sports concussion guidelines, what is a typical recovery period following a concussion, and what makes symptoms worse). There also were questions about parent perceptions concerning a student-athlete getting a concussion (e.g., what is the likelihood of your child getting a concussion, how concerned are you about your child getting a concussion, and what concussion symptoms concern you the most). There were additional questions about demographic variables and concussion history.

## Results

Knowledge questions

The survey included a variety of questions related to parental knowledge and perceptions regarding various aspects of concussion and its management. About 86% of participants had confidence (i.e., reasonably sure or sure) they could recognize a concussion in their child. In addition to parent reports of 11 children (6%) that had a diagnosed concussion, 10 others (5.6%) said that they suspected their child might have had a concussion (undiagnosed). In addition, 27% of respondents said that they observed their child get “dinged” or temporarily disoriented due to an impact to the head.

A majority of respondents (71.8%) agreed that a player should not return to play until he/she could exercise without experiencing symptoms. Yet, about one in five parents (22%) reported that they would allow their child to return to play after one week of rest. Most respondents (81.7%) reported that they were unaware of the Zurich guidelines [12] for sports concussion. A majority of respondents (73.9%) also reported no awareness of cognitive testing procedures often utilized in the evaluation and management of sports concussion. These last two issues would likely require specialized knowledge or experiences that most parents have not had.

Given the debate over how long a student-athlete should refrain from sports participation, we asked parents what they thought should be the typical amount of time necessary for recovery from a concussion (Table 1). While 113 (63.5%) gave conventional responses of days or weeks, 63 (35.4%) reported that it takes at least ‘months’ for recovery to typically occur and 12 respondents (6.7%) reported that no recovery would occur. Another interesting set of responses was generated by the question ‘which of the following activities would make concussion symptoms better or worse.’ There was recognition among most respondents that vigorous physical exercise could aggravate concussion symptoms (96.1%). However, there were also some surprising responses. For example, 86 respondents (47.8%) reported that ‘too much sleep’ would make concussion symptoms worse. Additionally, 46 respondents (25.6%) noted that ‘eating certain foods’ would do the same. Also, the sample was split on the activity of ‘studying,’ with 44.4% saying it could make concussion symptoms worse. An overall finding was that responses to a number of the questions were quite variable, suggesting a lack of uniformity and certainty in knowledge about concussions.

**Table 1:** Parents’ estimates of recovery time following concussion

Time	n (%)
Minutes	0 (0%)
Hours	2 (1.1%)
Days	40 (22.5%)
Weeks	73 (41.0 %)
Months	40 (22.5%)
Years	11 (6.2%)
No Recovery	12 (6.7%)

Perception questions

The survey asked respondents to answer various questions regarding concussion as it relates to their child playing football. One question asked how likely they thought it was that their child would get a concussion while playing football (Table 2). Overall, 166 respondents (92.2%) reported that it was at least ‘possible’ that their child would get a concussion with 25 (13.9%) reporting that it is ‘likely’ or ‘most likely.’ A follow-up question asked respondents to rate their concern about their child getting a concussion. The responses for this question showed high levels of concern with 176 of 178 (98.9%) reporting that they are at least ‘slightly concerned’ about the possibility and 46 (25.8%) saying that they are ‘quite concerned’ or ‘extremely concerned.’

**Table 2:** Parents’ estimates of concussion likelihood

Likelihood	n (%)
Never	2 (1.1%)
Unlikely	10 (5.5%)
Possibly	98 (54.4%)
Somewhat likely	45 (25.0 %)
Likely	21 (11.7%)
Most likely	4 (2.2%)

The last perception question on the survey asked respondents to rate their level of concern for each of 11 common symptoms/consequences of concussion (Table 3). The results from these ratings are both expected and unexpected. As expected, most concern was raised about potential brain damage as well as the possible long-term effects of the injury. High levels of concern were also noted for memory loss and loss of consciousness, both of which could suggest damage to the brain. Other concerns included academic problems and personality changes, two matters that would certainly affect overall adjustment and functioning within the family.

**Table 3:** Parents’ concerns about concussion symptoms

	Not at all concerned	Slightly concerned	Somewhat concerned	Quite concerned	Extremely concerned
Symptom	n (%)	n (%)	n (%)	n (%)	n (%)
Headache	3 (1.7%)	13 (7.3%)	50 (27.9%)	50 (27.9%)	63 (35.2%)
Loss of consciousness	3 (1.7%)	1 (0.6%)	15 (8.3%)	24 (13.3%)	137 (76.1%)
Memory loss	3 (1.7%)	2 (1.1%)	14 (7.8%)	22 (12.3%)	138 (77.1%)
Brain damage	2 (1.1 %)	3 (1.7%)	6 (3.4%)	19 (10.6%)	149 (83.2%)
Personality changes	4 (2.2%)	5 (2.8%)	18 (10.1%)	31 (17.3%)	121 (67.6%)
Academic problems	4 (2.2%)	4 (2.2%)	17 (9.5%)	33 (18.4%)	121 (67.6%)
Sleep difficulties	3 (1.7%)	4 (2.2%)	28 (15.6%)	55 (30.7%)	89 (49.7%)
Long-term effects	1 (0.6%)	3 (1.7%)	10 (5.6%)	19 (10.6%)	146 (81.6%)
Loss of playing time	69 (38.5%)	45 (25.1%)	20 (11.2%)	14 (7.8%)	31 (17.3%)
Nausea	3 (1.7%)	11 (6.1%)	43 (24.0%)	43 (24.0%)	79 (44.1%)
Losing position	105 (60.0%)	26 (14.6%)	16 (9.0%)	8 (4.5%)	23 (12.9%)

While respondents overall reported expected levels of concern for physical symptoms such as headaches and nausea, there were two surprising findings. There was a clear bimodal distribution of responses to two of the consequences of concussion, ‘loss of playing time’ and ‘losing position on the team,’ for which a subset of respondents reported ‘serious concern,’ 17.2% and 12.8%, respectively. It should be noted that there were no significant differences in the responses of mothers and fathers across all questions.

## Discussion

Youth football is popular, and not without risk, as youth players are just as likely to suffer a concussion or head impact as high school football players are [5]. Since there is usually no athletic trainer or other trained medical professional present at a youth football practice or game, coaches and parents have to make decisions about whether an athlete is injured and whether he or she should return to play, sit out, or seek medical attention [23]. Their decisions will be guided by what they know and believe about concussions, balanced against their motivation to have the injured child play football.

Most respondents in the current study felt that they could recognize a concussion in their child, were aware that most athletes recover in days or weeks, and were aware that athletes with a concussion cannot return to play if they cannot tolerate exercise without symptoms. By contrast, most had not heard of the Zurich guidelines [13] and were unaware of cognitive testing procedures that are now common in concussion evaluation and management, albeit mostly at the high school level and beyond. Respondents did have a good awareness of things that commonly aggravate concussion symptoms, including vigorous exercise, noise and commotion, and stress. However, a substantial number mistakenly believed that ‘too much sleep’ (48%) or ‘eating certain foods’ (26%) would make symptoms worse. Because we did not assess how parents acquired their knowledge, it is unclear how they arrived at these misconceptions. Nevertheless, it does demonstrate that parents of athletes still hold some misconceptions about brain injury [24].

The Zurich guidelines [13] specifically mention the need for ‘cognitive rest’ and ‘limiting scholastic activity’ in student athletes who have suffered a concussion. In our study, less than half (44%) of respondents correctly indicated that ‘studying’ could make concussion symptoms worse. Although the 'rest versus activity' debate continues, it appears that exertion, including mental over-exertion, may worsen symptoms and recovery, especially in the early stages of recovery [25]. Additionally, concussion symptoms that resolve at rest, but re-emerge when a child goes to school or does homework, may not be correctly attributed to the concussion. This may lead to a premature return to risky activities. There is some emerging evidence to suggest that even when symptoms resolve and there appears to be clinical recovery, the physical changes in the brain have not yet normalized [26]. Thus, we cannot be sure that return to play immediately after symptom abatement is totally safe. Students and parents will have more success managing symptoms if they understand what makes things worse, carefully monitor their child’s reactions to activities, consult their treatment professionals, and take a cautious, yet informed, management approach.

We found that parents do worry about the consequences of concussion. The majority of parents were most concerned about health outcomes, such as brain damage, loss of consciousness, and long-term effects like memory loss. Other significant concerns included academic and personality problems. All of these possible aftereffects could impact school performance and disrupt the lives of the student and family. In addition, a sizable group of parents seemed quite worried about their child’s possible loss of position or playing time. Worrying about playing time could promote a negative consequence of returning to play too soon, while unrealistic health worries might keep a child unnecessarily out of activities that he or she enjoys. Parents should have opportunities to talk with knowledgeable professionals about their concerns so that they can make informed, rather than reactive, decisions regarding when and what activities to allow.

Our survey indicated that 11 or approximately 6% of children sustained a concussion while playing youth football. While this incidence report aligns with other reported statistics [12], we should note that 10 other parents said they suspected their child sustained a concussion, while approximately half of the players were ‘shaken up’ at least once during the season and 27% were reportedly ‘dinged’ or had their ‘bell rung.’ This suggests that concussion is a viable concern in youth football, and that it may have been under-diagnosed or under-reported in this sample. Although the study design does not allow any firm conclusions about concussion incidence, Kroshus and colleagues [27] found that athletes have a tendency to under-report or fail to report symptoms in order to quickly return to play. Therefore, it is highly likely that our results underestimate the actual occurrence of concussions within this sample.

One explanation for under-reporting is that youth athletes feel pressure from coaches and parents to return to play before symptoms have completely resolved [27]. Educating parents on the signs, symptoms, consequences, and management of concussion could help decrease the pressure felt by athletes. The data from this current survey show that parents may be gaining knowledge from the increased public focus on concussion; however, information from the popular press may not accurately reflect a scientific understanding of concussion and its management, particularly as the science continues to emerge. Our results indicate that there is room for improvement in parental knowledge, including proper identification and management of symptomatic athletes [11-14]. Education about concussion may also reduce unnecessary anxiety regarding the risk of prolonged recovery or no recovery at all. Many parents seem to have fears related to concussions that are not warranted by research findings.

Limitations and directions for future research

This study allowed us to elicit information from a convenience sample of parents and guardians in central New York that permit children to play football. We are aware that this sample is relatively small and not representative of the entire population. Our findings may not generalize to other parents in other locales or to other sports, let alone to parents who do not have children playing contact sports. As with most surveys, we cannot be sure that the survey questions were clear and unambiguous, although these questions emerged from focus groups with parents. This survey investigated a person’s views at one moment in time. We realize that views on something like concussion are malleable. In fact, we recommend that investigators implement concussion education programs for parents and that parent knowledge and attitudes be assessed before and after the education program to determine the effectiveness of the training. The goal would not be to reduce the number of prospective athletes participating in scholastic sports but rather to promote informed, safe, and healthy sports decisions and player management.

## Conclusions

The data gathered in our study provide evidence of a need to improve the understanding of concussion and proper concussion management in the parents of youth football players and probably the parents of other young athletes as well. A substantial minority of parents in this study reported as much concern about playing time as about the symptoms of concussion, suggesting that both knowledge and attitudes toward concussion need to be targeted. We urge other organizations of youth sports (e.g., soccer, hockey, lacrosse, gymnastics, etc.) to undertake similar studies and continue to educate parents and staff about the evolving knowledge base related to sports concussion, and we urge schools and government agencies to take a leadership role in promoting such education among parents, coaches, trainers, and athletes.
